# Intake of Sugar-Sweetened Beverages in Adolescents from Troms, Norway—The Tromsø Study: *Fit Futures*

**DOI:** 10.3390/nu11020211

**Published:** 2019-01-22

**Authors:** Guri Skeie, Vårin Sandvær, Guri Grimnes

**Affiliations:** 1 Department of Community Medicine, UiT the Arctic University of Norway, N-9037 Tromsø, Norway; vsand@hotmail.com; 2 Nordland Fylkeskommune, Seksjon for Folkehelse, N-8048 Bodø, Norway; 3 Division of Internal Medicine, University Hospital of North Norway, N-9038 Tromsø, Norway; guri.grimnes@unn.no; 4 Tromsø Endocrine Research Group, Department of Clinical Medicine, UiT the Arctic University of Norway, N-9037 Tromsø, Norway

**Keywords:** adolescent, dietary behaviour, nutrition, Norway, sugar-sweetened beverages

## Abstract

High intake of sugar-sweetened beverages (SSB) has been associated with weight gain and chronic disease. The objective of this paper was to study the intake of SSB and characteristics associated with SSB intake in adolescents from Troms, Norway. We present results from a cross-sectional analysis from the Tromsø Study: *Fit Futures*, with 426 female and 444 male students aged 15–17 years (93% participation rate). Descriptive statistics and logistic regression analyses were performed. Among females, 31.8% drank at least one glass of SSB per day on average, compared to 61.0% among males. The adjusted OR (odds ratio) of daily SSB drinking for males vs. females was 3.74 (95% CI (confidence interval) 2.68–5.22). Other dietary habits such as eating snacks, drinking artificially sweetened beverages, fruit juice, and seldom eating breakfast were associated with higher odds for daily SSB drinking, as was daily snuffing. Weight class was not associated with daily SSB drinking. Students in vocational studies, particularly males tended to be more likely to be daily SSB drinkers. The prevalence of participants who on average were daily drinkers was higher than in national studies. We have identified several possible targets for interventions. Clustering of unhealthy behaviours and tendencies to socioeconomic differences are of particular concern.

## 1. Introduction

High intake of sugar-sweetened beverages (SSB) [1] has been associated with several health outcomes. Estimates from the Global Burden of Disease collaboration suggest that worldwide, 184,000 deaths per year are attributable to SSB consumption, mainly due to type 2 diabetes (*n* = 133,000), but also due to cardio-vascular diseases, and cancer [2]. A recent review on diabetes type 2 suggests a 13% higher incidence per serving per day, after adjustment for adiposity [3]. High sugar intake leads to dental decay [4], and SSB is one of the major sugar sources in many demographic groups, including Norwegian youths [5,6]. High SSB intake has also been associated with dental erosion, weight gain, and obesity, although the evidence is not unequivocal [7,8,9]. While it is the high sugar content that is associated with dental caries, the dental erosion is due to acidity [4,7], and therefore, replacement of SSB with light or artificially sweetened beverages might be beneficial for caries, but not have much effect on erosions. Dental caries has been the most common chronic disease of childhood [10], and damage to the permanent teeth due to caries or erosion, cannot be reverted.

The intake of SSB has been high in Norway, and the authorities have taken initiatives to reduce the intake [11,12]. In the period this study is covering, the aim was to reduce the number of daily consumers of SSB with 20% [13]. Over time, the trend in SSB intake among Norwegian children and adolescents has changed: Nationally representative cross-sectional studies performed in 1989–2001 as part of the international Health Behaviour in Among School-aged Children (HBSC) study, showed both a clear increase in frequency of intake of SSB and an increase in daily users over time [14]. More recently, from the 2005/06 survey, the reported intake has been lower, though not with a continuous decreasing trend [11,15]. In 2014, 11% of the males and 5% of the females drank SSB daily [16]. Another study comparing cross-sectional data from 11–13-year-old children before and after initiatives to reduce SSB consumption showed that the intake of lemonade and regular soft drinks, decreased, the intake of diet soft drinks increased, while juice consumption increased in males, and decreased in females [12].

A higher intake or frequency of intake, of SSB among males [12,15,17,18] and those with lower socio-economic status [12,17] is commonly reported, but in the HBSC study, there was no socio-economic difference in SSB consumption [11]. Some have questioned whether the association between SSB intake and type 2 diabetes is due to the sugar content of the SSB or related lifestyle factors such as other dietary practices, or (lack of) physical activity [19]. Intake of SSB has been associated with lifestyle factors such as physical activity and smoking [18]. Finally, consumption of SSB has been associated with parental modelling and regulation [20,21,22]. Earlier studies have suggested both poorer dietary habits among adolescents in Northern Norway [23], and found higher prevalence of dental caries as compared to southern parts of the country [24,25]. Our aim was to assess the proportion of daily consumers of SSB in 15–17-year-old adolescents from Troms, Norway, and their characteristics.

## 2. Material and Methods

The Tromsø study: *Fit Futures* is a population based longitudinal study with repeated measures of various indicators of lifestyle and health among adolescents [26]. The current paper is a cross-sectional analysis from the first survey in 2010–11, where all 1st grade students from all the eight upper secondary schools in Tromsø and Balsfjord municipalities were invited to participate. The catchment area includes both urban and rural populations. In 2010, 1301 students were enrolled as 1st year upper secondary school students, but 70 persons quit before *Fit Futures* 1 was conducted. Furthermore 114 students were sick or not reached for other reasons, leaving 1117 students who were invited to participate [26]. From those invited, 1038 students attended the study, giving a 93% participation rate.

Students were given time off from school and transported to the Clinical Research Unit of the University Hospital of North Norway, where trained personnel performed anthropometrical measurements, took blood samples, performed physical examinations, and conducted clinical interviews. The students completed self-administered digital questionnaires on a variety of health and lifestyle topics in addition to those detailed below. Information regarding school study programme was collected from school records. The Tromsø study: *Fit Futures* has been described in more detail elsewhere [26].

The age of the participants ranged between 15 and 28. Adolescents following a typical Norwegian educational progress are between the ages of 15–17 at 1st year of upper secondary school, and therefore students 18 years and older were excluded from the analyses (*n* = 77). Exclusions were also made for those missing data on variables used in the main analyses (*n* = 95), which therefore included 870 students, 426 females and 444 males.

The Norwegian Data Protection Authorities and the Regional Committee for Medical and Health Research Ethics have approved the *Fit Futures* study (ref no 2009/1282 and 2012/1904). The study was conducted in accordance with the Declaration of Helsinki. All participants in *Fit Futures* signed an informed consent declaration. For students under the age of 16 additional written consent was provided by their guardians.

### 2.1. Dietary Variables

The questionnaire assessed consumption frequency of 14 different foods/food-groups and 10 different beverages. The questions on diet can be found in supplementary file 1. Questions regarding beverages had the response categories “seldom/never”, “1–6 glasses per week”, “1 glass per day”, “2–3 glasses per day” and “4 glasses or more per day”. The replies to the two questions on sugar-sweetened carbonated and non-carbonated soft drinks were summed based on the category midpoints (e.g., if they answered 2–3 glasses per day on both questions, it would be counted as 5 glasses per day). A binary variable was constructed, distinguishing between those who, on average, drank SSB daily and not. Similarly, the two questions on light/artificially sweetened carbonated and non-carbonated beverages were combined into one, as were the four questions on milk/yoghurt. The new variables were categorized in order to avoid low cell counts.

For fruits and vegetables (two questions), the categories were “seldom/never”, “1–3 times per month”, “1–3 times per week”, “4–6 times per week”, “1–2 times per day”, “3–4 times per day” and “5 times per day”. These variables were combined into one variable for number of fruits and vegetables eaten per day. This variable was not normally distributed, and therefore recoded into a categorical variable with four categories, based on the distribution. The variables for sweets (e.g., chocolate and drops) and sweet and savoury snacks (e.g., potato crisps, cakes, cookies, buns) had the same categories as the fruit and vegetables, except “every day” was the highest frequency. Again, categories were combined on a-variable-to-variable basis in order to avoid low cell counts.

We assessed frequency of eating breakfast, dinner (the main hot meal), and lunch. As Norwegian students are not served meals at school, those who do not bring their lunch from home (usually sandwiches) often buy something to eat at some nearby store/kiosk, or possibly a canteen. Therefore, bringing lunch from home could be a marker of a healthier diet, or stronger parental control. For breakfast and dinner, the response categories were “every day”, 4–6 days a week”, “1–3 days a week” and “seldom/never”. For lunch brought to school, categories were “every school day”, “3–4 days a week” “1–2 days a week”, and “seldom/never”. Breakfast and lunch were recoded into three categories covering seldom/never, most days, and every (school) day. Dinner was recoded into every day/not every day.

### 2.2. Other Variables

Study program was classified as general studies (including sports and physical education studies) and vocational studies. We combined the information about physical activity from two questions: First, the students indicated whether or not they were doing sports or physical activity (such as skateboarding, soccer, dancing or running) outside school hours. Then, weekly sports/physical activity outside school hours was assessed with the original categories: “none”, “about 30 min”, “about 30–90 min”, “about 2–3 h”, “about 4–6 h”, “7 h or more”. Those who on the first question indicated no activity were combined with the lowest group on the second question and coded none. The next three categories for weekly activity outside school hours were coded as up to three h a week, and the last two as 4 h or more.

Students were asked about average time spent in front of computers, TV, DVD or similar outside of school hours, differentiating between school days and weekend days. Categories were the same as for physical activity, except for the highest categories: “about 7–9 h” or “10 h or more”. School days and weekend days were weighted 5/7 and 2/7 respectively and combined into one variable. The variable daily screen time was split into categories of “<2 h”, “2–3.99 h” and “≥4 h” per day.

Students were asked about smoking and snuff (or snus, a form of smokeless tobacco, a moist powder tobacco placed under the upper lip) habits, with response categories “no, never”, “sometimes” or “daily”. Due to few daily smokers, they were combined with occasional smokers. Students were asked how often they drank alcohol, with options “never”, “once per month or less”, “2–4 times per month”, “2–3 times per week” or “4 times or more per week”. The highest categories had few responders and were combined into the category “2 times per month or more”.

A question on who the students lived with (some form of family guardian, friends or alone) was used to differentiate between those who had moved out of home and not. Students answering that they lived both with parents and had moved out of home (*n* = 7) were assumed to be commuters and were therefore included in the group that had moved out of home. Only two students answered living in an institution (and having moved out of home). They were still included in the group living at home, as it is likely that an institution would have an adult in charge of the food environment. 

BMI was calculated as weight in kg divided by the square of height in meters (kg/m^2^) and Cole and Lobstein’s revised gender- and age-specific cut-offs [27] were used for classification of BMI categories. Due to few thin and obese students, these groups were merged with the categories normal weight and overweight, respectively.

Students were asked about parents’ educational level. The categories “primary school” and “upper secondary school” were combined due to few responders. Furthermore, this variable differentiated between higher education for less and more than four years. The category “do not know” was combined with “missing”. Since around 25% of the students did not know or did not answer the questions on mother’s and father’s education, the variables were only used for additional analyses, and not introduced in the main statistical model.

### 2.3. Statistical Analyses 

Descriptive and analytic statistical analyses were performed using SAS version 9.4 (SAS Institute Inc., Cary, NC, USA). Two-sided *p*-values < 0.05 were considered statistically significant. As the variables included in the analyses were categorical, unadjusted analyses were performed using contingency tables with chi-square tests, with the percentage of students that on average reported daily drinking of SSB as outcome. Data from all participants were used to calculate the percentage daily drinkers, but the numbers for non-daily drinkers have been suppressed for increased legibility. Adjusted analyses of characteristics associated with daily drinking of SSB were performed using logistic regression. We considered health related characteristics (smoking, using snuff, drinking alcohol, physical activity, screen time, body mass index), school programme, whether the student lived with parents/guardians, dietary variables (fruits and vegetables, sweets, snacks, beverages other than SSB, and meals eaten) as potential covariates. As there was a large difference in SSB drinking between sexes, both unadjusted and adjusted analyses were performed for each sex separately. However, in order to compare the results obtained between sexes, the adjusted logistic regression model was constructed by including independent variables which were associated with daily drinking of SSB (*p* < 0.25) in the unadjusted analyses of the total sample, weighted for sex. Variables were manually excluded until only significant covariates remained in the model. An additional model was constructed including also sex, mother’s and father’s education, in order to estimate the effect of sex, and study socio-economic differences from an additional perspective. Father’s education was not a significant covariate and was not included in the final analyses. To assess potential sex differences in the associations, we fitted interaction terms with sex, and re-ran the regression analyses for each covariate.

No indication of multicollinearity was observed when evaluating the variance inflation factor. Hosmer and Lemeshow’s goodness-of-fit test was used to evaluate overall fit of the regression models. To evaluate how much of the variance in daily drinking of SSB the model explained, pseudo *R*^2^ (Nagelkerke/Cragg and Uhler’s) was assessed.

## 3. Results

The mean age of the participants was 16.1 year (Table 1). While most females were in general study programs (61.7%), most males had chosen vocational studies (55.0%). Most students lived with their parents/guardian (females 85.9% and males 87.8%). More males (61.3%) than females (32.2%) reported drinking SSB on average every day (*p* < 0.001). Females tended to drink water more frequently than males, while males tended to drink milk more often than females. About half of the students had breakfast daily, and about one-third brought lunch to school every school day, females slightly more often than males. Almost 60% of the females and 73% of the males had dinner daily.

The prevalence of overweight/obesity was high, 19.3% in females and 23.7% in males (Table 1). A larger proportion of males than females was in the group with the highest screen time (47.7% vs. 35.0%). Regarding physical activity outside school hours, the distribution in females and males seemed to be similar. While 20.0% of females and 23.4% of males smoked occasionally or daily, 32.9% of females and 39.6% of males used snuff occasionally or daily. The proportion of students reporting never drinking alcohol was quite low (females 23.5%, males 32.7%).

**Figure 1:**
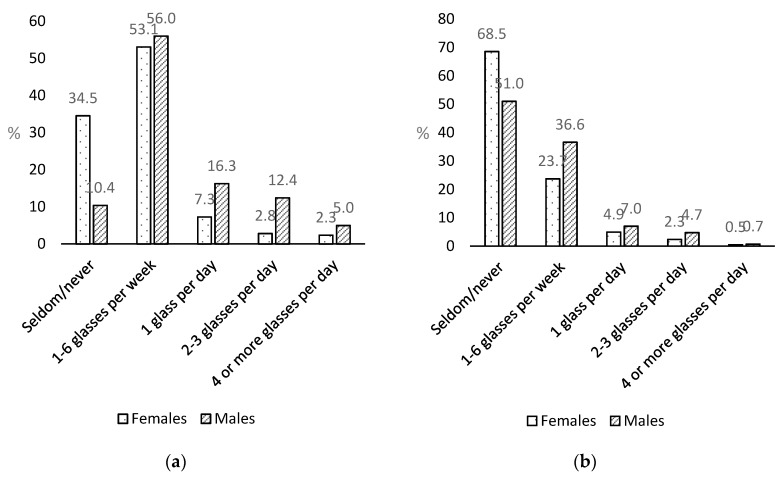
Intake of (**a**) carbonated sugar-sweetened beverages (SSB); and (**b**) non-carbonated SSB, by sex.

More than 50% of the females and males drank carbonated SSB 1–6 times a week, but more males than females drank them daily (Figure 1a). Non-carbonated SSB was drunk less frequently than carbonated SSB, and the majority reported drinking them seldom/never, though males also consumed these beverages more often than females (Figure 1b).

In univariate analyses, living with parents/guardians was not associated with daily SSB drinking, neither in females nor in males (Table 2). Almost 70% of males in vocational studies were daily drinkers, compared to half of the males in general studies. In females the corresponding percentages were slightly less than 50 in vocational studies and just above 20 in general studies (*p* < 0.001 in both sexes).

Among females eating less than 0.6 servings of fruits and vegetables daily, there were 40.2% daily drinkers of SSB, compared to 27.1% among those eating 2.3 servings or more (*p* = 0.008). The corresponding numbers for males were 69.5% and 57.0% (*p* = 0.05). Both among males and females, those who drank a glass or more of light/artificially sweetened beverages or fruit juice daily, also had a higher tendency to drink SSB daily. There were differences in prevalence of daily drinkers of SSB of 20–30 percentage points between the highest and lowest categories of these beverages for both sexes. Similar, but smaller, differences were found for more frequent consumption of sweets and snacks. In both sexes, the higher the intake of water, the lower the percentage of daily SSB drinkers (*p* < 0.02). There was no association between drinking SSB and milk consumption.

Rarely eating breakfast or bringing lunch to school was associated with daily SSB drinking, with 20–40% higher prevalence of daily SSB drinking than among those eating these meals every day (Table 2). No association was found between frequency of having dinner and daily SSB drinking. Weight class (thin/normal vs. overweight/obese) was not associated with daily SSB drinking, neither in females nor males. Those not physically active were more often daily drinkers of SSB than the more active, though the distributions differed between sexes, and the difference was only borderline significant in males. Higher daily screen time was associated with daily SSB drinking in females (*p* = 0.002), but not in males. Smokers and snuff users were more likely to be daily SSB drinkers. Among males, never drinkers of alcohol were less likely to be daily SSB drinkers than more frequent alcohol drinkers, among females, there was no significant association between alcohol drinking and daily SSB drinking in univariate analyses.

In the adjusted model, students in vocational studies had a 59% higher chance of drinking SSB daily compared to those in general studies (only borderline significant for females) (Table 3). Daily drinking of SSB was associated with several dietary variables: eating snacks weekly vs. not (females OR 3.67, 95%CI 2.03–6.64, males OR 2.03, 95% CI 1.29–3.19), drinking light/artificially sweetened beverages (>1 glass daily vs never—females OR 4.05, 95%CI 2.22–7.36, males OR 2.20, 95% CI 1.30–3.72). The same was seen for fruit juice, those drinking a glass or more daily had an odds ratio of daily SSB drinking of 2 or more, compared to never drinkers (significant for both sexes). Not eating breakfast was associated with higher odds of daily drinking of SSB compared to eating everyday (females OR 4.03, 95% CI 1.88–8.63, males OR 2.02, 95% CI 0.97–4.22). Other health behaviours were also associated with daily SSB drinking: A significant association with daily snuffing (females OR 4.93, 95% CI 2.40–10.14, males OR 2.18, 95% CI 1.14–4.16), and the same tendency, although not statistically significant, was seen for daily/occasional smoking. Never drinkers of alcohol had a higher OR of daily SSB drinking than those drinking alcohol twice a month or more often, at least in females (females OR 2.59, 95% CI 1.19–5.66, males OR 1.60, 95% CI 0.84–3.03). Water consumption was no longer a significant covariate. The adjusted model explained 40.8% of the variation in daily drinking of sugar-sweetened beverages for females and 20.6% for males. However, there was no interaction with sex (all *p*-values for interaction >0.08). 

**Figure 2:**
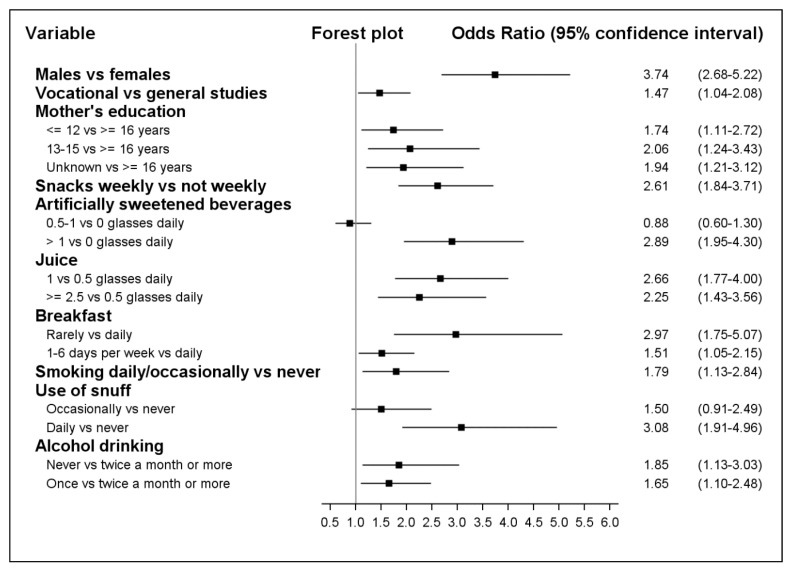
Odds for being a daily drinker of sugar-sweetened beverages in the Tromsø study: *Fit Futures*. Mutually adjusted model including sex and mother’s education, *n* = 870.

In the model including sex and mother’s education, males had 3.74 times higher odds of being daily SSB drinkers compared to females (95% CI 2.68–5.22) (Figure 2). Compared to students with mothers with the longest education, those with mothers with short (OR 1.74, 95% CI 1.11–2.72) or medium length of education (OR 2.06, 95% CI 1.24–3.43), had higher odds of being daily SSB drinkers. Else the results were similar to the main model, with OR estimates between what was found for females and males, but estimates for smoking and alcohol were stabilized. This model explained 37.6% of the variation in daily SSB drinking. There was no interaction with sex, (all *p*-values for interaction >0.12).

## 4. Discussion

We found high prevalence of daily SSB drinking in this group of adolescents from Northern Norway. Socio-economic factors (study programme, mother’s education), sex, diet (snacks, breakfast), other drinking habits (light/artificially sweetened beverages, fruit juice), and health-related habits (smoking, snuffing, alcohol drinking) all contributed to explaining the variation in daily SSB drinking. The model explained more of the variation in daily SSB drinking in females than in males.

In our study, there were more than twice as many daily drinkers as in the edition of HBSC that took place in the same period and age group, 61% of the males and 32% of the females vs. 25 and 11% [11,15]. We included two questions (carbonated and non-carbonated SSB) rather than one, but it is not likely that this, and differences in response categories, explains the difference in prevalence, but rather that there are differences in intake between the two studies. The HBSC study included a lower number of students from the Northern region, but they used a cluster sampling to ensure a nationally representative sample [11], how this might have influenced the results is uncertain. A previous study from Northern Norway found that among boys, as many as 52.3% of eighth graders and 74.0% of tenth graders were daily drinkers of carbonated SSB, corresponding numbers for girls were 42.1% and 46.8% [23]. In addition, 48.9% of the boys and 45.0% of the girls drank other soft drinks (“squash”) daily. That survey took place five years before our study. As we found that 61% of the males and 32% of the females were daily drinkers (of carbonated and non-carbonated SSB combined), this might support the decreasing trend in SSB intake seen over time in the HBSC study [11]. However, the number of daily drinkers still is higher than what is reported in national studies and studies from other parts of Norway. Some of this difference can be explained by differences in age groups and questions included in the different studies, but not all. While our questions covered more than HBSC, we did not include energy/sports drinks, so our prevalence estimates could still be underestimated. Our participants represented urban and rural adolescents in Northern Norway. We excluded some students due to age, and some due to missing variables, but with an exceptionally high participation rate, we still think that the results are representative for adolescents in the region.

Conflicting results exists concerning the stability of SSB consumption. The Oslo Youth survey found moderate to high stability of soft drink consumption from age 15 to 25, but low stability from age 15 to 33 [18]. The FVMM study on the other hand observed a decrease in SSB consumption, and an increase in consumption of artificially sweetened beverages from age 11 to 26 [28]. Hence, continued monitoring of our participants would be interesting in order to gain more knowledge about the stability of the SSB intake.

Higher or more frequent intake in boys than in girls has been reported in several studies [17,29,30,31,32]. However, some of the other results were surprising; we did not see associations with BMI, sedentary behaviour, or physical activity in the final models. A lack of association between BMI and consumption of SSB has also been reported in other studies [29]. One study found no association in cross-sectional analyses, but higher BMI in SSB consumers in longitudinal analyses [30]. The same study found associations both cross-sectionally and longitudinally for percentage body fat. A review on confectionery consumption and overweight/obesity found that in cross-sectional studies, obese and overweight children had lower confectionary intakes than normal weight children [33]. In addition, they reported no association in longitudinal studies. Our study is a cross-sectional study, so no causal inferences can be drawn. It is possible that those with high BMI underreport their SSB consumption, or actually reduce their consumption in order to lose weight [29]. Alternatively, BMI might not be the best measure of body fatness, or this could be a true association. Spending much time on sedentary activities could also mean spending much time on homework, and need not be only a negative feature.

One could assume that more frequent consumption of one drink would be mirrored by lower/less frequent consumption of other drinks, but daily drinking of SSB was associated with higher consumption of juice. The picture was more mixed for artificially sweetened soft drinks, however, daily drinkers were more frequently never consumers of alcohol, at least among females. In the univariate analyses, there was less SSB consumption among those that drank water more frequently. This association was not significant in the adjusted analyses. Replacing SSB with water would be the healthiest option, but interventions have only produced medium size increases in water consumption [6]. Longitudinal analyses of stability and change across the spectrum of beverages would provide interesting complimentary data. Those rarely eating breakfast had a very high odds for daily SSB-drinking. For this group, availability of healthy options in schools might be an issue.

A recent publication with data from the Nordic countries in the HBSC study found no socioeconomic differences in SSB drinking, as measured by the family affluence scale [11], however, our study suggested that both own educational choice and mother’s education were associated with daily SSB drinking, in line with other studies [12,17,20,28,29,30,32,34]. To the best of our knowledge, this is the first Norwegian study to look at school programme, an indicator of the students own socioeconomic status, in association with SSB consumption. If longitudinal studies can confirm that school programme is a predictor of SSB consumption, targeted interventions in vocational school programmes might be an important avenue for further reducing the SSB consumption. Not living with parents did not influence SSB drinking, but the group was small, so no strong conclusions can be drawn.

The HELENA study assessed the beverage consumption among European adolescents in eight countries [31]. SSB was the second most frequently consumed beverage, after only water, and 55% of the 15–17.5 year olds reported drinking SSB (including sports drinks) during the two days the study covered, compared with our 61% daily drinkers among males and 32% among females. In half the countries studied, SSB were the largest contributor to energy intake among all beverages.

SSB are major contributors to sugar intake in Norwegian adolescents [35], and dietary patterns characterized by high sugar and fat content have been associated with adiposity later in adolescence [36]. The current national plan of action for a better diet aims at halving the number of 15-year-olds that drink SSB 5 times a week or more often [37]. Continued monitoring, and interventions targeting SSB consumption in general, and in males, students in vocational programmes and Northern Norway in particular are warranted, in order to accomplish that goal. Reviews have suggested that education programmes and changes in school environment (restricted access) will reduce intake, but also that substitution of SSB with low sugar/artificially sweetened beverages will reduce sugar and energy intake [38,39]. This is in line with children’s own opinions [40]. Restricting access at home and ensuring good parental modelling is another possible avenue [20,22,38,40]. A recent review showed only small decreases in SSB consumption in adolescents after interventions [6], specific analyses on most successful intervention settings could not be done in adolescents, but for children, home-based interventions and modelling/demonstrating the behaviour seemed to improve intervention effects. 

This study has several strengths, a fairly large, population-based sample, a very high participation rate, and assessment of a range of covariates that might confound analyses of SSB intake and health. The sample was homogenous with respect to age, and included both rural and urban participants. Taken together, this suggests that our results are representative for adolescents in Northern Norway. Separate questions were asked for both carbonated and non-carbonated SSB, as this better captures the range of products that can be grouped as SSB [41].

The study also has several limitations. The questions about SSB used in this study have not been used in other studies in adolescents and, in general, most of the studies cited here have used different definitions or response categories, hampering comparisons between studies. For instance, not all studies distinguish between carbonated and non-carbonated SSB, and many do not include sports drinks (and neither did ours). Not including sports drinks/energy drinks in the definition probably means that frequency of intake reported here is underestimated. Lack of comparability is a recognized problem, and a recent review has suggested methods that should be used in future studies [41].

The most frequently reported intake category in our study was wide (1–6 glasses per week), and created possibilities for misclassification when combining the data for carbonated and non-carbonated SSB into one variable. Some studies distinguish weekday and weekend consumption, as this may differ considerably [17], but we did not have that information. Consequently, some of the participants we labelled “daily SSB drinkers” might have consumed larger amounts of SSB on one or two days, rather than one (or more) glass daily. The total amount of sugar and energy ingested will be the same, independent of how the consumption is distributed, but the length of time the teeth are exposed will affect dental caries risk.

Furthermore, the food/drink questions have not been validated. A review on validity of food frequency questionnaires used in adolescents showed that not assessing portion size, shorter time span, medium length, and administration directly to the adolescent, not via a parent gave the best validity [42]. Our questionnaire did not specify the time-span for the food questions, but the other factors were fulfilled. Our study only included participants from Northern Norway, and the results might not be representative for the rest of the country.

As cutting down on sugar intake has been official policy during the last years, and initiatives have been taken to reduce consumption of SSB [13], social desirability bias, i.e., underreporting consumption of products the health authorities discourage, could be an issue. Given the high consumption reported, this seems less likely, but it cannot be ruled out. Although the sample was relatively large, the skewed distribution of some of the variables led to large uncertainty in some of the estimates. The study was cross-sectional, so it is not possible to make causal inferences. For some reason, the variables explained the variation in SSB intake in females better than in males. We lacked information regarding, e.g., individual choices vs. family or peer influence, and availability, or money to purchase SSB [20,21,34,40]. Perhaps such variables are more important for males’ food choices than females’. Females reach puberty earlier and have a stronger body focus, and possibly also health focus than males [43,44], this may explain some of the sex differences we found.

## 5. Conclusions

In conclusion, particularly males are frequent drinkers of SSB, despite efforts at reducing consumption in recent years. In both males and females, higher prevalence of daily drinking was found than in a national survey. Our study has identified several factors associated with daily SSB drinking, many of them are other unhealthy lifestyle choices, and are important to adjust for in analyses of SSB intake and health. If these associations are confirmed using stronger study designs, these factors could be targeted together in future comprehensive intervention studies. The higher consumption of SSB in vocational studies is of particular concern, suggesting that socio-economic differences in diet start early.

## Figures and Tables

**Table 1:** Characteristics ^1^ of the participants in the Tromsø study: *Fit Futures.*

	Females (*n* = 426)	Males (*n* = 444)
Age, years	16.1	16.1
Living with parent(s)	85.9	87.8
Mother’s education		
Elementary or secondary school	34.3	32.0
College/university < 4 years	19.0	16.4
College/university 4 years or more	23.5	22.3
Unknown/missing	23.2	29.3
Father’s education		
Elementary or secondary school	38.7	39.0
College/university < 4 years	13.4	13.5
College/university 4 years or more	18.8	18.2
Unknown/missing	29.1	29.3
General study programme (including sports)	61.7	45.1
Drink sugar-sweetened beverages daily	32.2	61.3
Servings of fruits and vegetables, daily		
Less than 0.6	22.8	34.7
0.6–1.3	18.1	22.3
1.4–2.3	26.3	22.1
More than 2.3	32.8	20.9
Consume chocolate/sweets at least weekly	70.2	68.5
Consume sweet or savoury snacks at least weekly	64.3	68.0
Light/artificially sweetened beverages, daily		
None	40.6	43.9
0.5 glass	32.6	26.6
1 glass or more	26.8	29.5
Fruit juice, daily		
0.5 glass or less	65.5	63.7
1 glass	19.2	21.4
2.5 glasses or more	15.3	14.9
Milk, daily ^2^		
1 glass or less	42.7	40.6
1.5–2.5 glass	31.5	26.1
2.5 glasses or more	25.8	33.3
Water, daily		
1 glass or less	18.3	31.1
2–3 glasses	34.5	33.8
4 or more glasses	47.2	35.1
Breakfast		
Seldom/never	15.0	12.2
1–3 days per week	35.2	35.3
Every day	49.8	52.5
Bring lunch for school		
Seldom/never	23.0	34.9
1–6 days/week	43.4	39.0
Every school day	33.6	26.1
Dinner daily	58.5	73.4
Overweight/obese	19.3	23.7
Screen time, daily ^3^		
<2 h	26.3	14.0
2–3.99 h	38.7	38.3
4 h or more	35.0	47.7
Physical activity outside school hours, weekly		
Not active	30.0	33.3
3 h or less	30.3	26.6
4 h or more	39.7	40.1
Smoking daily or occasionally	20.0	23.4
Use of snuff		
Never	67.1	60.4
Occasionally	14.3	12.8
Daily	18.6	26.8
Alcohol drinking		
Never	23.5	32.7
Once per month or less often	46.9	37.1
Twice per month or more often	29.6	30.2

^1^ Mean age, otherwise percentage distributions. ^2^ Sum of four different milk types (different fat content) and yoghurt. Includes both plain and fermented milk. ^3^ Weighted mean of weekends and weekdays.

**Table 2:** Daily drinkers of sugar-sweetened beverages according to demographic, dietary and lifestyle characteristics, by sex in the Tromsø study: *Fit Futures*.

Characteristic	Females (*n* = 426) ^1^		Males (*n* = 444) ^1^	
	Daily Drinkers % (n ^2^)	*p*-Value ^3^	Daily Drinkers % (n ^2^)	*p*-Value ^3^
Living with parent(s)		0.63		0.84
Yes	32.6 (119/365)		61.4 (239/389)	
No	29.5 (18/61)		60.0 (33/55)	
School programme		<0.0001		0.0003
Vocational	46.6 (76/163)		68.9 (168/244)	
General (including sports)	23.2 (61/263)		52.0 (104/200)	
Servings of fruits and vegetables, daily		0.008		0.05
Less than 0.6	40.2 (39/97)		69.5 (107/154)	
0.6–1.3	42.9 (33/77)		60.6 (60/99)	
1.4–2.3	24.1 (27/112)		53.1 (52/98)	
More than 2.3	27.1 (38/140)		57.0 (53/93)	
Consumption of chocolate/sweets		0.007		0.007
Not weekly	22.8 (29/127)		52.1 (73/140)	
Weekly	36.1 (108/299)		65.5 (199/304)	
Consumption of sweet or savoury snacks		<0.0001		0.004
Not weekly	18.4 (28/152)		51.4 (73/142)	
Weekly	39.8 (109/274)		65.9 (199/302)	
Light/artificially sweetened beverages, daily		<0.0001		0.003
None	24.3 (42/173)		54.4 (106/195)	
0.5 glass	20.9 (29/139)		56.8 (67/118)	
1 glass or more	57.9 (66/114)		75.6 (99/131)	
Fruit juice, daily		0.0006		0.002
0.5 glass	25.8 (72/279)		55.1 (156/283)	
1 glass	43.9 (36/82)		72.6 (69/95)	
2.5 glasses or more	44.6 (29/65)		71.2 (47/66)	
Milk, daily ^4^		0.28		0.10
1 glass or less	34.6 (63/182)		67.2 (121/180)	
1.5–2.5 glass	26.9 (36/134)		56.0 (65/116)	
2.5 glasses or more	34.6 (38/110)		58.1 (86/148)	
Water, daily		0.02		0.0005
1 glass or less	41.0 (32/78)		74.6 (103/138)	
2–3 glasses	36.1 (53/147)		56.0 (84/150)	
4 glasses or more	25.9 (52/201)		54.5 (85/156)	
Breakfast		<0.0001		0.002
Seldom/never	67.2 (43/64)		74.1 (40/54)	
1–6 days/week	31.3 (47/150)		68.2 (107/157)	
Every day	22.2 (47/212)		53.7 (125/233)	
Bring lunch to school		<0.0001		0.0006
Seldom/never	50.0 (49/98)		68.4 (106/155)	
1–4 days/week	31.4 (58/185)		64.7 (112/173)	
Every school day	21.0 (30/143)		46.6 (54/116)	
Dinner		0.09		0.21
Not daily	36.7 (65/177)		66.1 (78/118)	
Daily	28.9 (72/249)		59.5 (194/326)	
Weight class		0.38		0.59
Thin/normal weight	33.1 (114/344)		62.0 (210/339)	
Overweight/obese	28.1 (23/82)		59.1 (62/105)	
Screen time, daily ^5^		0.002		0.24
<2 h	22.3 (25/112)		54.8 (34/62)	
2–3.99 h	29.7 (49/165)		58.8 (100/170)	
4 h or more	42.3 (63/149)		65.1 (138/212)	
Physical activity outside school hours, weekly		0.0001		0.06
Not active	46.9 (60/128)		65.5 (97/148)	
3 h or less	26.4 (34/129)		66.1 (78/118)	
4 h or more	25.4 (43/169)		54.5 (97/178)	
Smoking		< 0.0001		0.005
Never	26.7 (91/341)		57.7 (196/340)	
Occasionally or daily	54.1 (46/85)		73.1 (76/104)	
Use of snuff		< 0.0001		0.001
Never	23.1 (66/286)		54.9 (147/268)	
Occasionally	39.3 (24/61)		63.2 (36/57)	
Daily	59.5 (47/79)		74.8 (89/119)	
Alcohol drinking		0.1		0.02
Never	29.0 (29/100)		52.4 (76/145)	
Once a month or less often	29.0 (58/200)		67.9 (112/165)	
Twice a month or more often	39.7 (50/126)		62.7 (84/134)	

^1^ All participants are used to calculate the percentage of daily drinkers, but the numbers for the non-daily drinkers have been suppressed for better legibility. ^2^ The n is the number of daily drinkers/the total number of participants reporting the given characteristic. ^3^ Chi-square test. ^4^ Sum of four different milk types (different fat content) and yoghurt. Includes both plain and fermented milk. ^5^ Weighted mean of weekends and weekdays.

**Table 3:** Characteristics Associated with being a daily drinker of sugar-sweetened beverages in the Tromsø study: *Fit Futures*.

	Females (*n* = 426)		Males (*n* = 444)	
Characteristic	OR	95% CI	OR	95% CI
School programme				
General	Ref		Ref	
Vocational	1.59	(0.94–2.68)	1.59	(1.03–2.46)
Consumption of sweet or savoury snacks				
Not weekly	Ref		Ref	
Weekly	3.67	(2.03–6.64)	2.03	(1.29–3.19)
Light/artificially sweetened beverages, daily				
None	Ref		Ref	
0.5 glass	0.77	(0.41–1.46)	0.99	(0.60–1.64)
1 glass or more	4.05	(2.22–7.36)	2.20	(1.30–3.72)
Fruit juice, daily				
0.5 glass or less	Ref		Ref	
1 glass	2.12	(1.13–3.95)	2.82	(1.62–4.92)
2.5 glasses or more	2.34	(1.18–4.64)	2.13	(1.13–4.03)
Breakfast				
Every day	Ref		Ref	
1–6 days/week	1.19	(0.68–2.08)	1.68	(1.04–2.70)
Seldom/never	4.03	(1.88–8.63)	2.02	(0.97–4.22)
Smoking				
Never	Ref		Ref	
Occasionally or daily	1.92	(0.98–3.76)	1.59	(0.84–2.99)
Use of snuff				
Never	Ref		Ref	
Occasionally	2.35	(1.12–4.91)	1.14	(0.56–2.30)
Daily	4.93	(2.40–10.14)	2.18	(1.14–4.16)
Alcohol drinking				
Twice a month or more often	Ref		Ref	
Once a month or less often	1.39	(0.76–2.54)	2.00	(1.13–3.53)
Never	2.59	(1.19–5.66)	1.60	(0.84–3.03)

Mutually adjusted logistic regression analyses. Ref = reference category, OR = odds ratio, CI = confidence interval.

## Supplementary Materials

The following are available online at http://www.mdpi.com/2072-6643/11/2/211/s1, Supplementary File 1: Dietary questions used in the Tromsø study: *Fit Futures 1*.

## Abbreviations

BMI	Body mass index (kg/m^2^)
CI	Confidence interval
HBSC	Health Behaviour among School Children study
OR	Odds ratio
SSB	Sugar-sweetened beverages
