# Supplementary material for: Intake of Sugar-Sweetened Beverages in Adolescents from Troms, Norway—The Tromsø Study: Fit Futures
Source: Nutrients. 2019 Jan 22;11(2):211. doi: 10.3390/nu11020211 (PMC6412924; doi:10.3390/nu11020211)
Supplement: Supplementary file 1 [file nutrients-11-00211-s001.pdf]

FORHÅNDSVISNING

FF - Generelt spørreskjema - Uke 1

MATVANER OG KOSTHOLD

66) Hvor ofte pleier du å spise følgende i løpet av en uke?

	Hver dag	4-6 dager i uka	1-3 dager i uka	Sjelden eller aldri
Frokost	○	○	○	○
Middag	○	○	○	○

67) Hvor ofte spiser du matpakke hjemmefra på skolen?

- ○ Hver dag
- ○ 3-4 ganger per uke
- ○ 1-2 ganger per uke
- ○ Sjelden eller aldri

68) Hvor ofte spiser du vanligvis disse matvarene?

	Sjelden/ aldri	1-3 ganger per måned	1-3 ganger per uke	4-6 ganger per uke	Hver dag
Ost (alle typer)	○	○	○	○	○
Fet fisk (f.eks. laks, ørret, makrell, sild)	○	○	○	○	○
Mager fisk (f.eks. torsk, sei, hyse)	○	○	○	○	○
Pizza, hamburger eller pølser	○	○	○	○	○
Hermetisert mat (fra metallbokser)	○	○	○	○	○
Godteri (f.eks. sjokolade, drops)	○	○	○	○	○
Snacks og søtsaker (f.eks. potetgull, kake, kjeks, bolle)	○	○	○	○	○
Sukkerfri tyggegummi	○	○	○	○	○

<< Tilbake | Neste >>

58 % completed

© Copyright www.questback.com. All Rights Reserved.

FORHÅNDSVISNING

FF - Generelt spørreskjema - Uke 1

69) Hvor ofte spiser du vanligvis

	Sjelden/ aldri	1-3 ganger per mnd	1-3 ganger per uke	4-6 ganger per uke	1-2 ganger per dag	3-4 ganger per dag	5 eller flere ganger per dag
Frukt	○	○	○	○	○	○	○
Grønnsaker	○	○	○	○	○	○	○

70) Hvor mange ganger i året spiser du vanligvis disse matvarene?

	0	1-3	4-5	6-9	10 eller flere
Mølje med fiskelever	○	○	○	○	○
Måsegg	○	○	○	○	○
Reinsdyrkjøtt	○	○	○	○	○
Selvplukket sopp	○	○	○	○	○

<< Tilbake | Neste >>

59 % completed

© Copyright www.questback.com. All Rights Reserved.

FORHÅNDSVISNING

FF - Generelt spørreskjema - Uke 1

71) Hvor mye drikker du vanligvis av følgende?

	Sjelden/ aldri	1-6 glass per uke	1 glass per dag	2-3 glass per dag	4 glass eller mer per dag
Helmelk, kefir, yoghurt	○	○	○	○	○
Lettmelk, cultura, lettyoghurt	○	○	○	○	○
Skummet melk (sur/søt)	○	○	○	○	○
Ekstra lett melk	○	○	○	○	○
Juice	○	○	○	○	○
Saft med sukker	○	○	○	○	○
Lettsaft, kunstig søtet	○	○	○	○	○
Brus med sukker (1/2 liters flaske = 2 glass)	○	○	○	○	○
Lettbrus, kunstig søtet (1/2 liters flaske = 2 glass)	○	○	○	○	○
Vann	○	○	○	○	○

72) Bruker du følgende kosttilskudd?

	Ja, daglig	Iblant	Nei
Tran, trankapsler, fiskeoljekapsler	○	○	○
Vitamin- og/eller mineraltilskudd	○	○	○

<< Tilbake | Neste >>

61 % completed

© Copyright www.questback.com. All Rights Reserved.
